# Amoxicillin-docosahexaenoic acid encapsulated chitosan-alginate nanoparticles as a delivery system with enhanced biocidal activities against *Helicobacter pylori* and improved ulcer healing

**DOI:** 10.3389/fmicb.2023.1083330

**Published:** 2023-02-09

**Authors:** Saeed Khoshnood, Babak Negahdari, Vahab Hassan Kaviar, Nourkhoda Sadeghifard, Mohd Azmuddin Abdullah, Mohamed El-Shazly, Mohammad Hossein Haddadi

**Affiliations:** ^1^Clinical Microbiology Research Centre, Ilam University of Medical Sciences, Ilam, Iran; ^2^Department of Medical Biotechnology, School of Advanced Technologies in Medicine, Tehran University of Medical Sciences, Tehran, Iran; ^3^Department of Toxicology, Advanced Medical and Dental Institute, Universiti Sains Malaysia, Penang, Malaysia; ^4^Department of Pharmacognosy, Faculty of Pharmacy, Ain-Shams University, Cairo, Egypt

**Keywords:** chitosan-DHA nanoparticles, *Helicobacter pylori*, biocidal activities, amoxicillin, encapsulation, chitosan, docosahexaenoic

## Abstract

Encapsulation of amoxicillin (AMX) for drug delivery against *Helicobacter pylori* infection and aspirin-induced ulcers in rat’s stomachs was performed using docosahexaenoic acid (DHA)-loaded chitosan/alginate (CA) nanoparticles (NPs) developed by ionotropic gelation method. The physicochemical analyses of the composite NPs were performed by scanning electron microscopy, Fourier transform infrared spectroscopy, zeta potential, X-ray diffraction, and atomic force microscopy. The encapsulation efficiency of AMX was increased to 76% by incorporating DHA, which resulted in a reduction in the particle size. The formed CA-DHA-AMX NPs effectively adhered to the bacteria and rat gastric mucosa. Their antibacterial properties were more potent than those of the single AMX and CA-DHA NPs as demonstrated by the *in vivo* assay. The composite NPs attained higher mucoadhesive potential during food intake than during fasting (*p* = 0.029). At 10 and 20 mg/kg AMX, the CA-AMX-DHA showed more potent activities against *H. pylori* than the CA-AMX, CA-DHA, and single AMX. The *in vivo* study showed that the effective dose of AMX was lower when DHA was included, indicating better drug delivery and stability of the encapsulated AMX. Both mucosal thickening and ulcer index were significantly higher in the groups receiving CA-DHA-AMX than in the groups receiving CA-AMX and single AMX. The presence of DHA declines the pro-inflammatory cytokines including IL-1β, IL-6, and IL-17A. The synergistic effects of AMX and the CA-DHA formulation increased the biocidal activities against *H. pylori* infection and improved ulcer healing properties.

## Introduction

Stomach infection with *Helicobacter pylori*, a Gram-negative bacterium with carcinogenic potential, is a major cause of gastric malignancies such as MALT lymphoma and gastric adenocarcinoma (Kim and Wang, 2021). Successful *H. pylori* eradication could reduce the risk of metachronous gastric cancer by 50% (Choi et al., 2020). In the majority of cases (89.4%), the first line of treatment against *H. pylori* infection is clarithromycin (CLA) and amoxicillin (AMX) or metronidazole (MET) in combination with a proton pump inhibitor (PPI) (Suzuki and Matsuzaki, 2018). However, antibiotic resistance emerged in recent years and gastric acid was found to inactivate some antibacterial agents (Qin et al., 2021). As the clinical isolates of *H. pylori* have become increasingly resistant to antibiotics worldwide, there is an urgent need to improve therapeutic strategies with more effective antibiotic regimens to reduce treatment failures (Krzyżek et al., 2020).

*H. pylori* showed resistance to MET and CLA and a less degree to AMX (Graham et al., 2021). Therefore, AMX is considered the most reliable antibiotic therapy in almost all regimens (Kuo et al., 2021). Stomach acid however can decompose and destroy AMX, reducing its overall and local effectiveness. There are two approaches to overcome this limitation including intravenous administration for at least 7 days or the oral administration of high doses of AMX (Shah et al., 2021). The use of nanopolymers such as chitosan to encapsulate acid-sensitive drugs could protect AMX from degradation by gastric acid, prolong their retention/residence time, and improve its controlled release (Spósito et al., 2021). Chitosan is a cationic mucoadhesive biopolymer, and based on molecular weight (MW), can be divided into high, medium, and low MW, each having different biological properties. High MW chitosan containing high degrees of deacetylation is superior to low MW chitosan for the treatment of *H. pylori*. Moreover, chitosan exhibited more potent antibacterial activity against Gram-negative bacteria than Gram-positive bacteria and this effect was attributed to the stronger negative charge on the cell walls of the Gram-negative bacteria (Takahashi et al., 2008; Li et al., 2016; Chang et al., 2020).

Fatty acids (FAs) that exhibit antibacterial activity against multidrug-resistant (MDR) bacteria could provide the next generation of antibacterial agents for the treatment and prevention of bacterial infections (Coraça-Huber et al., 2021). A combination of FAs and antibiotics was tested suggesting their potential application in the treatment of bacterial resistance. The combination of FAs with beta-lactam antibiotics, fluoroquinolones, and aminoglycosides showed a synergistic effect against Gram-positive and Gram-negative bacteria (Casillas-Vargas et al., 2021). Docosahexaenoic acid (DHA) is one of the omega-3 polyunsaturated fatty acids (PUFAs) with anti-inflammatory and anti-*H. pylori* properties. In the presence of DHA, *H. pylori* expand its periplasmic space, resulting in the loss of membrane integrity, cytoplasmic leakage, and cell death (Correia et al., 2012). To date, there is only one report addressing the anti-*H. pylori* activity of the encapsulated DHA when administered through nanostructured lipid carriers (Seabra et al., 2017). Chitosan-based nanoparticles (NPs) possess mucoadhesive properties rendering them interesting candidates to be tested as enhancers of the antibacterial effect of AMX and DHA. The present study aimed to develop a chitosan-based oral drug delivery system containing DHA and AMX against *H. pylori*, both *in vitro* and *in vivo.* Chitosan/alginate (CA) composite NPs were used to entrap AMX and DHA. The physicochemical characterizations of the NPs were assessed. The antibacterial activity was determined using an *in vitro* growth inhibition assay, followed by the evaluation of the mucoadhesive potential of the FITC-labeled NPs. In the *in vivo* experiments, an aspirin-induced gastric ulcer was induced in rats and the rats were infected with *H. pylori*. To determine the biocidal effects against *H. pylori* and the curing effect of the composite NPs on the induced ulcer, the *H. pylori* colonization, ulcer area, and histopathological changes were monitored.

## Materials and methods

In the Supplementary material, more details are provided regarding the materials and methods used in this study. The materials are described in the Supplementary section 1.

### Fabrication of composite NPs and formulation

A CA-based NP was prepared based on a previous study by Friedman et al. (2013). Composite NPs were prepared by emulsifying a chitosan solution in an oil phase (DHA) and an ionic gelation method. Chitosan and alginate solutions were prepared as polycationic and polyanionic solutions (Supplementary section 2).

CA-DHA NPs were prepared in several formulations including chitosan (0.1, 0.5, and 1.0% v/v), DHA (0.0, 0.5, 1.5, and 2.0% v/v), and AMX (40, 60, and 100 mg/ml). The *in vivo* study was conducted to test CA-DHA, CA-AMX, and CA-DHA-AMX NPs with the following concentrations of the components: CA (1.0% v/v), DHA (2.0% v/v), and AMX at two concentrations (10 and 20 mg/kg).

### Physicochemical characteristics

The physicochemical properties of the composite NPs were evaluated using a scanning electron microscope (SEM), Fourier transforms infrared spectroscopy (FTIR), X-ray diffraction (XRD), atomic force microscopy (AFM), zeta potential, and swelling index (SI) analysis, and the methods are described in detail in Supplementary section 3.

### Drug content

The content of DHA and AMX in the recovered solution was measured using a UV/Vis spectrophotometer (JENWAY/6105) at 205 and 272 nm, respectively. To compare the loading of AMX with the hydrophobic and hydrophilic compounds, it was tested in the emulsion and aqueous environments. A detailed explanation of the method used to determine the drug content can be found at Supplementary section 3.

### Antibacterial activity

The growth inhibitory assay was used to determine the antibacterial activity against clinically isolated *H. pylori* (H.12.5) *in vitro*. Briefly, 10 μl of the bacterial suspension (10^9^ CFU/ml) was added to Columbia broth medium (190 μl) and the mixture was incubated under microaerophilic conditions for 6, 12, and 24 h. The growth inhibition assay was performed *in vitro*. Growth inhibition was estimated from the absorbance of the medium at a wavelength of 600 nm. Supplementary section 4 describes the patient’s details, the method of bacterial isolation, the *in vitro* antibacterial assay, and the formula for growth inhibition. To evaluate antibacterial activity *in vivo*, plate colony counts (CFU/gr stomach) and the number of bacteria in the biopsy samples were used (Supplementary section 4).

### Bacterial binding and mucoadhesive activity

Bacterial adhesion was measured using FITC-labeled composite NPs (Supplementary section 5). The adhesion was determined by flow cytometry and was subsequently analyzed using the FlowJo program (Tree Star). The bacterial binding and mucoadhesive activity were conducted on the four composite NPs including unloaded NPs, CA-DHA with three different DHA concentrations, CA-AMX, and CA-DHA-AMX. The adhesion rate of NPs to the bacterial cell surface was determined at 2 and 4 h intervals.

The mucoadhesive activity was evaluated using the count method according to the previous method of Arora et al. on rat stomachs (Arora et al., 2011). The mucoadhesive potential of NPs was calculated by fluorescence microscopy using the following formula:


$$\;\mathrm{Mucoadhesive}\%=\frac{\left(\mathrm{Cs}-\mathrm{Cd}\right)}{\mathrm{Cd}}\times 100$$

The input and output counts of NPs are represented by “Cs” and “Cd,” respectively.

The *in vivo* study was performed with NPs on the mucosa in two different nutritional states including the fasting and fed states. ImageJ v1.52 software (NIH, United States) was used to calculate the fluorescence intensity of the adherent FITC-labeled NPs.

### Animals

One hundred forty-seven male Sprague Dawley rats were involved in this study. Six rats were lost during infection and ulcer induction. Eighteen stomachs from rats were used to evaluate the *in vitro* mucoadhesive study*. In vivo* mucoadhesive activity was performed for two main feeding conditions: fasting and fed. Each of the two groups consisted of four different sub-groups (CA, CA-DHA, CA-AMX, and CA-DHA-AMX). A total of 24 rats were used to determine the mucoadhesive potential *in vivo*.

In the infected groups, rats were fasted overnight before being treated with 250 mg/kg body weight (BW) acetylsalicylic acid (ASA) on day 0 to induce gastric ulcers. Bacterial infection was induced by oral administration of *H. pylori* (5 × 10^8^–10 CFU/ml, 1 ml/rat) at 24, 48, and 72 h after ASA ulceration. Infection was confirmed after 2 weeks, and treatment was initiated. Three rats were used to confirm *H. pylori* colonization and three rats were used for the gastric ulcer confirmation. The *in vivo* antibacterial activity of different formulations was assessed in thirty rats divided into ten groups at day 7 post-infection (Supplementary Tables S1, S2).

The *in vivo* studies were performed to investigate the ulcer healing activity by macroscopic and microscopic analysis of gastric ulcers by ulcer thickness, ulcer area, ulcer index, and *H. pylori* eradication was also determined by the direct bacterial counting method. Collagen accumulation and concentrations of inflammatory cytokines, including IL-1β, IL-6, and IL-17A, were determined in the treated and untreated groups on day 14 post-infection. Forty-two rats were randomly divided into seven groups (six rats/group); group 1 (NS, normal saline), groups 2 and 3: AMX (powder 10 and 20 mg/kg WB), groups 4 and 5: CA-AMX (10 and 20 mg/kg WB), and groups 6 and 7: CA-DHA-AMX (10 and 20 mg/kg WB) to evaluate ulcer healing and *H. pylori* eradication on day 14. Eighteen rats were divided into six treated groups to evaluate the relapse of infection on day 21 post-infection (three rats/group). One uninfected group was defined as control (*n* = 3).

We performed a histopathological examination on gastric biopsies. The ulcer evaluation was done on the whole stomach on day 14 to assess the healing effect. Ulcer indexing was performed according to the previous study by Bhattacharya et al. (2006). To measure the ulcer area, the length and thickness of the ulcer in mm^2^ were measured at a magnification of 40× and the information was processed using IimageJ software. The area of the gastric ulcer in each section (5 sections/sample) was determined. Gastric histology was evaluated by 1 pathologist who was blinded to the other assays and results.

All animal experiments were performed according to accordance with the U.K. Animals (Scientific Procedures) Act, and protocol approved by the Ethics Committee of Ilam Medical University (approval number: R.MEDILAM. REC.1400.133).

### Enzyme-linked immunosorbent assay

A whole blood sample was taken from the rats. The presence of IL-1β, IL-6, and IL-17A in serum was measured using ELISA kits according to the manufacturer’s instructions. The intensity was determined at 450 nm using a microplate reader. To ensure consistency of the assay, all plates contained positive control (FBS) and negative control (PBS) samples.

### Masson’s trichrome staining and immunohistochemistry

Masson’s trichrome staining, which stains collagen fibers blue and faint green, was used to assess collagen accumulation. Naturally, collagen fibers are usually accumulated in the gastric mucosa and submucosa. Immunohistochemistry was performed using antibodies against type I collagen. Sections were incubated with 0.3% hydrogen peroxide in PBS for 30 min and then with 10% normal donkey or goat serum in 0.05 M PBS for 30 min. They were then incubated with polyclonal antibodies labeling collagen I. Each sample was imaged using a Nikon TE 2000 fluorescence microscope (Nikon, Japan). The area of collagen in each section was measured using ImageJ software.

### Statistical analysis

The obtained results were statistically analyzed using Mann–Whitney’s *t*-test with a confidence level of 95% (*p* < 0.05). Statistical analysis was performed using the Wilcoxon Sum Rank test for the ulcer index. Differences between means were tested for more than two groups with a one-way analysis of variance (ANOVA) followed by a Bonferroni’s *post-hoc* test. Differences between groups and time points were analyzed with a two-way analysis of variance (ANOVA) and subsequent multiple comparisons using the Bonferroni correction. *p < 0.05; **p < 0.001; ***p = 0.0002; and ****p < 0.0001 indicated statistically significant results.

## Results and discussion

### Composite nanoparticle production

This study showed that the NPs were formed by the interactions between the positively charged amino groups of the polycationic agent (chitosan) and the negatively charged polyanionic agent (alginate). Based on the strong mucoadhesive properties of CA NPs (Chang et al., 2021), we developed a CA-based NP containing DHA and AMX to test their ulcer healing and *H. pylori* eradication properties. Based on the *in vitro* results, we tested the physicochemical properties of CA-DHA-AMX at the defined concentrations of 1% v/v, 2% v/v, and 60 mg/ml, respectively.

**Figure 1:**
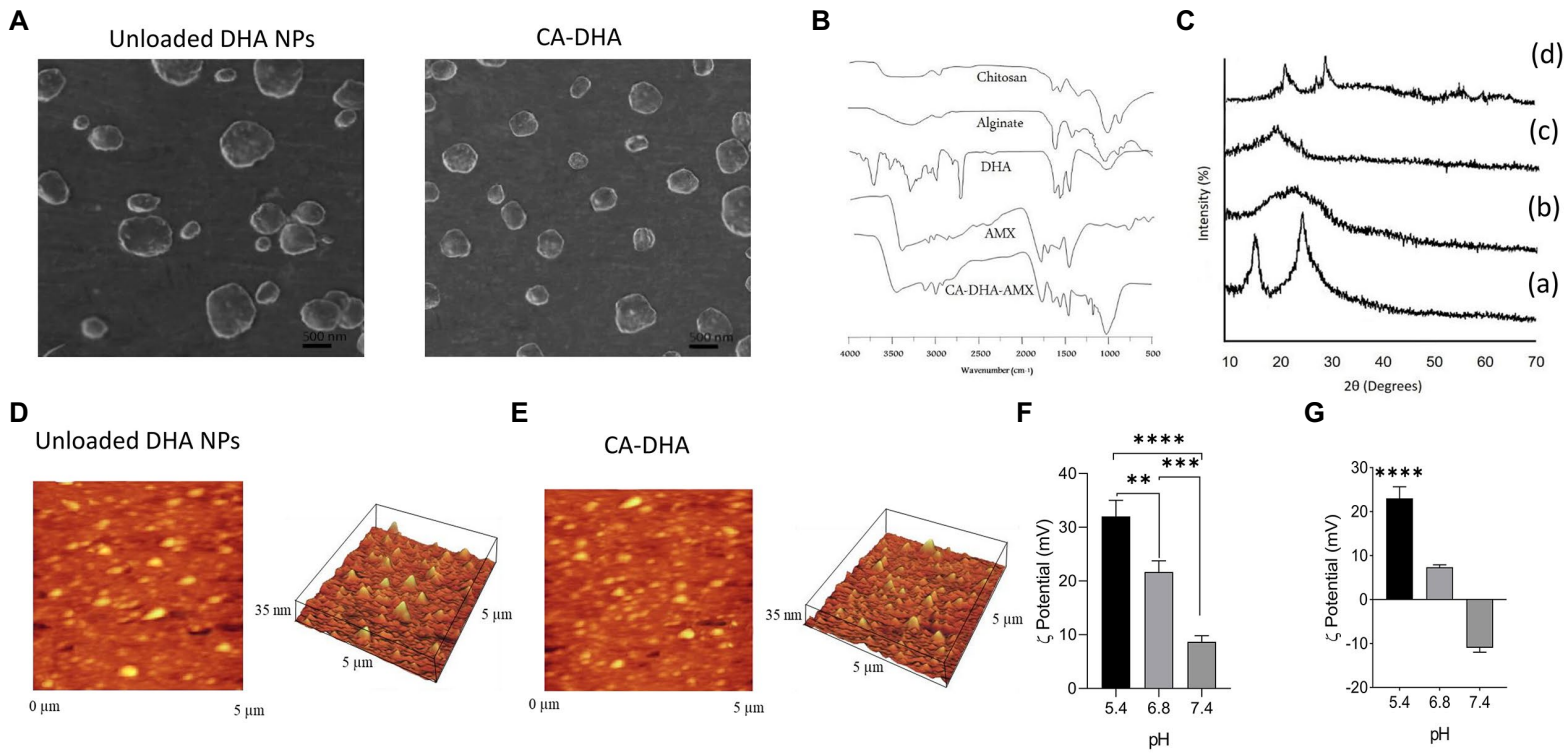
Physicochemical properties of CA-based NPs. **(A)** SEM micrograph of CA NPs and CA-DHA-AMX NPs (CA = 1%, DHA = 100 μM, 2% v/v, and AMX = 60 mg/ml). **(B)** FTIR spectra of chitosan, alginate, DHA (100 μM), AMX, and CA-DHA-AMX NP. **(C)** XRD patterns of chitosan (a), alginate (b), DHA (c), and CA-DHA (d). **(D,E)** AFM analysis of the surface topography of the unloaded and DHA-loaded NPs. **(F,G)** Zeta potentials of unloaded and DHA-loaded NPs (2% v/v) at various pH levels.

As demonstrated by SEM and FAM, the composite nanoparticles with DHA produced smaller NPs (350 ± 110 nm) with a smoother surface than the DHA-free NPs (600 ± 92 nm) in a concentration-dependent manner. Their spherical shape is due to a hydrophobic group (DHA) in their structure. NPs with smaller sizes were obtained during the oil-in-water micelle structure, resulting in a homogeneous dispersion of DHA (Figure 1A).

FTIR spectra of the composite NP CA-DHA-AMX and their components are shown in Figure 1B. The CA-DHA showed a significantly intensified peak at 1679 cm^−1^, while the peaks of the amino group at 1596 cm^−1^ and carboxyl groups at 1619 cm^−1^ disappeared. An ionic interaction was confirmed between the carboxyl group of the alginates and the amino group of chitosan. The new major peak at 1750 cm^−1^ showed a significant increase in absorption confirming the presence of DHA in CA-based scaffolds. AMX major peaks were observed at 3,440 cm^−1^ (amide NH and phenol OH stretch), 3,020 cm^−1^ (benzene ring C-H stretch), 1,770 cm^−1^ (beta-lactam C-O stretch), 1,680 cm^−1^ (amide I C-O stretch), 1,500 cm^−1^ (benzene ring C-C stretch), and 1,480 cm^−1^ (N-H bend C-N stretch combination band). Characteristic peaks of AMX were also present in the FTIR spectrum of the composite beads with some broadening and reduction in intensity, indicating the absence of the chemical interactions between the drug, polymer, and counter ions after the formation of beads.

As shown in Figure 1C, the pattern of chitosan showed two wide typical diffraction peaks at 2θ = 13.5° and 25.5°, confirming the semicrystalline nature of this molecule, which are the hydrated and anhydrous polymorphs of chitosan, respectively. The XRD spectrum of alginate showed a typical wide crystalline peak at 2θ = 21.5°. No crystalline peak was observed for DHA. The peaks of chitosan and alginate disappeared, and two new peaks appeared at 2θ = 23.4° and 27.5° in the XRD spectrum of the CA-DHA scaffold, confirming the presence of DHA in the structure of the new scaffold. Strong interactions between the chitosan amino groups and the alginate cation groups were related to the changes in the membrane crystallinity. The ionic interaction between chitosan and alginate significantly decreased the crystallinity of the scaffold, indicating its amorphous state (Cui et al., 2008).

AFM analysis revealed that the synthesized NPs were almost monodisperse, without agglomeration (Figures 1D,E). Evidence suggested that particle aggregation decreased in the presence of hydrophobic agents (Dixon et al., 2012; Chang et al., 2021). In this study, the presence of DHA in CA-DHA formulations resulted in the reduction of particle aggregation. The particle size distribution was more homogeneous in the presence of 100 μM DHA than for the unloaded NPs. The variation of the zeta potential of the composite NPs was mainly due to the negative charge of the NP-loaded polymers. Compared with CA-DHA, the unloaded NPs exhibited a more positive charge, supporting the presence of the charged DHA in the NPs. The surface charges of the composite NPs were directly affected by the pH changes in the dispersion medium, and the highest value was observed at pH 5.4 (Figures 1F,G).

In a composite polymer, swelling occurs due to water absorption, causing the NPs to expand during water penetration, which leads to an increased crosslinking of the composite polymers (Pacheco et al., 2019). DHA concentration, the acidity of the medium, and time exhibit a direct effect on the rate of water uptake by NPs. The protonation of the carboxylic groups of the alginate increased at an acidic pH (pH < 4), resulting in shrinkage of the polymer due to the reduction of electrostatic repulsion between these hydrophobic groups (Rahaiee et al., 2017). This study showed a satisfactory correlation between the swelling indexes, is decreased by the addition of an extra hydrophobic group such as DHA (Supplementary Figure S1). A favorable agreement was observed in comparison with a previous study by Chang et al. who reported that the hydrophobicity of cobia liver oil (CBLO) decreased the swelling and aggregation of chitosan resulting in smaller particle size (Chang et al., 2021). At all pH, composite NPs were soluble after a 6 h adjustment (data not shown).

An influential factor that affects the effectiveness of drug encapsulation is the effectiveness of drug entrapment. The drug entrapment efficacy of DHA and AMX was also evaluated in the same way as shown in Table 1. The highest drug content was obtained at pH 5.4 in the chitosan solution, 65 ± 4% and 71 ± 1.9% for DHA and AMX, respectively. Also, when DHA was included in the CA-DHA-AMX formulation, the drug content increased.

**Table 1:** The efficacy of NPs at entrapping DHA and AMX in different pH levels of the dispersion medium.

The pH of dispersion medium	Composite NPs			
	CA-DHA	CA-AMX	CA-DHA-AMX	
	DHA %	AMX %	DHA %	AMX %
5.4	65 ± 4	71 ± 1.9	63 ± 2.6	76 ± 5.7
6.8	62 ± 3.2	58 ± 1.9	64 ± 2.1	62 ± 3
7.4	51 ± 1.8	49 ± 5.3	47 ± 3.6	50 ± 6.2

### Antibacterial activity

**Figure 2:**
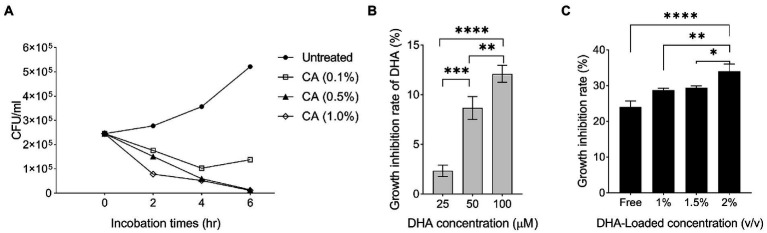
The antibacterial activity of DHA and chitosan alone and in combination. **(A)** The growth inhibition rate of CA NPs with different concentrations of chitosan (0.1, 0.5, and 1.0%). **(B)** The antibacterial activity of DHA with different concentrations (25, 50 and 100 μM). **(C)** The antibacterial activity of DHA -loaded NPs at different loaded concentrations of DHA (CA 0.1%, DHA 1, 1.5 and 2%).

Previous reports indicated that DHA exhibited a potent antibacterial effect on *H. pylori* and inhibited its colonization in animal models (Correia et al., 2012; Ji et al., 2016; Henriques et al., 2020). In this study, the unloaded NPs with a chitosan concentration of 1.0% exhibited higher antibacterial activity than the NPs at other concentrations (*p* < 0001; Geisser–Greenhouse’s epsilon = 0.510; Figure 2A). This result supported the findings of Luo et al. who reported that a high degree of deacetylation (95%) of chitosan exhibited stronger antibacterial activity than deacetylation of 88% (Luo et al., 2009). In our study, the antibacterial activity of DHA was the highest at 100 μM (Figure 2B). We formulated CA-DHA at a concentration of 1.0% v/v CA and three different concentrations of DHA, including 1, 1.5, and 2% v/v. The bacterial growth was strongly suppressed at 2% v/v DHA (Figure 2C).

**Figure 3:**
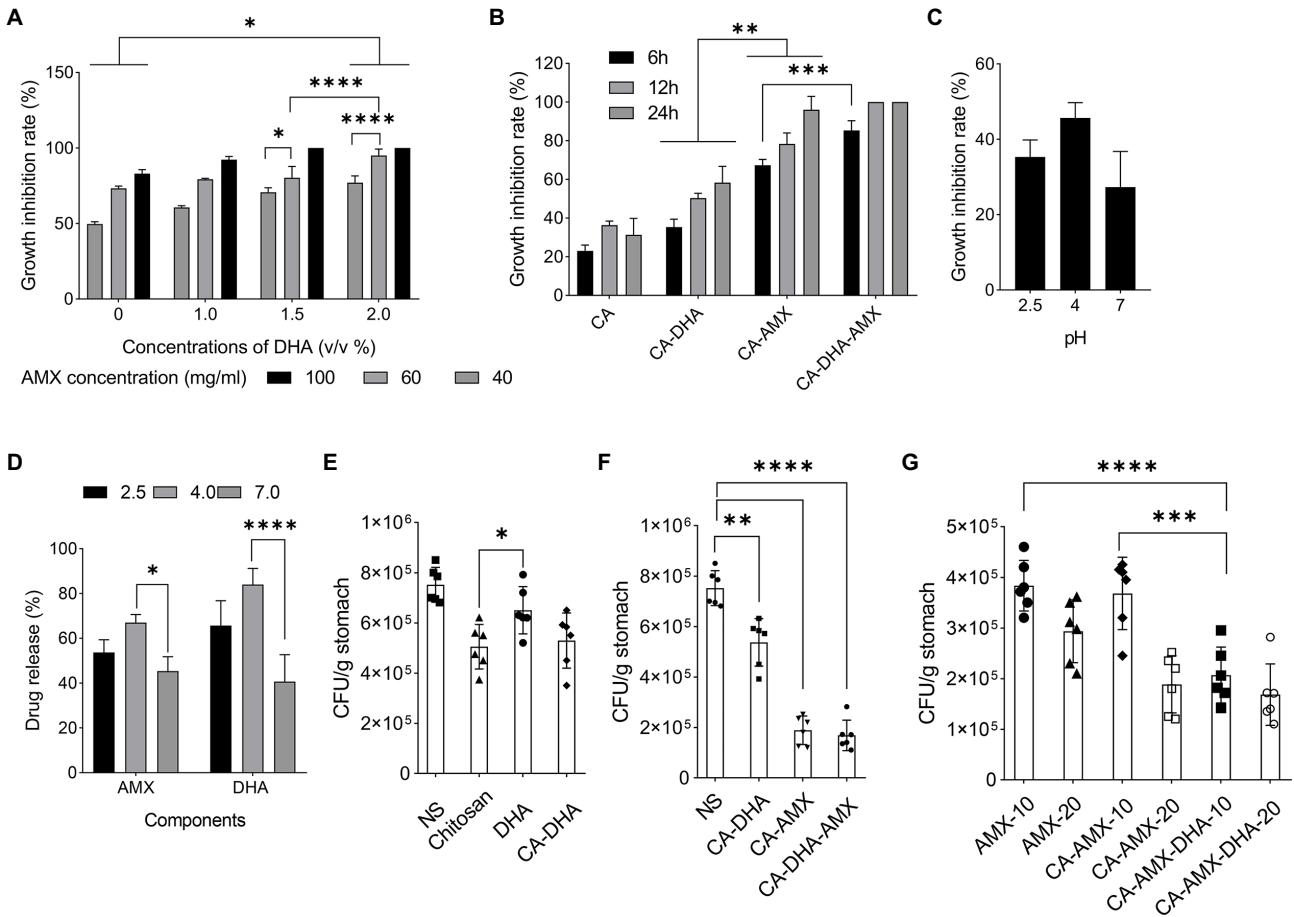
The growth inhibition activity of composite NPs. **(A)** The antibacterial activity of CA-NPs containing different concentrations of DHA and AMX. **(B)** The growth inhibition rate of CA-DHA (100 μM)-AMX (60 mg/ml) composite NPs at different time intervals 6, 12, and 24 h. **(C,D)** CA-DHA (100 μM)-AMX (60 mg/ml) at different pH values after 6 h incubation was investigated for their growth inhibition activity and release of drugs. **(E)** The *in vivo* antibacterial activity of chitosan, DHA and CA-DHA. **(F)** *In vivo* antibacterial activity of various formulations of composite NPs, including CA-DHA, CA-AMX, and CA-DHA-AMX compared with the untreated group. **(G)** Antibacterial activity was tested at a dose of 10 and 20 mg/kg AMX in different formulations. Data are presented as mean ± SEM.

Previous results showed that the encapsulation of DHA increased its antibacterial activity. To improve the antibacterial efficacy against *H. pylori*, previous research examined the nanoencapsulation of DHA alone (Seabra et al., 2017). In this study, DHA and AMX were incorporated into CA-based NPs to maximize drug delivery and reduce antibiotic concentration. The results showed that CA-DHA with 2% v/v DHA significantly increased the growth inhibition rates compared with the unloaded NPs (*p =* 0.013) (Figure 3A). According to previous studies, DHA-loaded NPs showed bactericidal activity against *H. pylori* but not against human gastric adenocarcinoma cells at bactericidal concentrations (Chang et al., 2020; Henriques et al., 2020). We also observed a significant difference between the two concentrations of DHA (1.5 and 2% v/v; *p* < 0.0001). Therefore, we examined the composite NPs with 2% v/v DHA. CA-DHA-AMX proved to be more effective against bacteria when the dose of AMX was increased (Figure 3A). Thus, we conducted further studies with the following formulation: CA (1% v/v)- DHA (2% v/v)-AMX (60 mg/ml). After 6 h of incubation, it was found that the composite NP exhibited a synergistic antibacterial effect when DHA and AMX were used together (Figure 3B). Despite the significantly higher antibacterial activity of CA-AMX NPs than CA-DHA NPs (*p =* 0.0042), the presence of DHA in the CA-DHA-AMX formulation significantly increased antibacterial activity (*p =* 0.0009) (Figure 3B).

After a diet, the pH of the stomach changes within 4–6 h. The acidity of the stomach is 2.5 during starvation, while it increases to 3.0–4.5 during feeding (Chen et al., 2008). Potent antibacterial activity was observed in CA-DHA-AMX at pH 4 (Figure 3C). Hu et al. reported that the antibacterial activity of chitosan decreased at pH > 5.8 (Hu et al., 2007). Among the tested groups, CA-DHA-AMX showed minimal growth activity, probably due to AMX and DHA. The low antibacterial activity in an acidic medium arises from the degradation of AMX, while at pH 7.0, only a small amount of the active ingredient is released from the NPs. Our study was conducted to determine how much of the active ingredient is released after 6 h at different pH values. The release of AMX and DHA at pH 4.0 was comparable to the release at pH 2.5, but the release at pH 7.0 was significantly less than the release at pH 4.0 (pH 4.0 vs. 7.0; *p =* 0.029 and > 0.001 for the release of AMX and DHA, respectively) (Figure 3D).

Compared with DHA, both CA-DHA and pure chitosan showed higher antibacterial activity *in vivo*. This suggested that the antibacterial properties of NPs may be derived from their chitosan coating (*p =* 0.04) (Figure 3E). The lower antibacterial activity of DHA can be attributed to its anionic charge and lipophobic content, while the bactericidal activity can be attributed to the electrostatic interactions between the cationic charges (NH_2_; pH < 6) of chitosan and the polyanionic acid of the bacterial cell wall, leading to membrane disintegration, osmotic pressure disturbance, and eventually cell death (Chandrasekaran et al., 2020).

Significantly increased antibacterial activity was observed in the groups with AMX (Figure 3F). We investigated the effects of encapsulation of AMX and incorporating DHA on drug delivery and eradication of *H. pylori* by comparing the antibacterial activities of AMX, CA-AMX, and CA-DHA-AMX. In contrast to the free AMX, the encapsulated AMX showed a significant enhancement of antibacterial activity. Based on the results of this study, there was an additive effect of DHA in CA-DHA-AMX on the antibacterial activity of 10 mg/kg AMX (Figure 3G). Compared with CA-AMX, CA-DHA-AMX showed more potent antibacterial activity. Even when the AMX concentration decreased, the antibacterial activity increased more in the CA-DHA-AMX group than in the CA-AMX group (20 to 10 mg/kg) (Figure 3G). This increase might be related to the better localization and accessibility of the drug (Arora et al., 2011). Another possible explanation is that the treatment of DHA alters the composition of the membrane proteins and bacterial cell walls (Correia et al., 2013).

The results showed that the most potent antibacterial activity in CA-DHA-AMX was at 20 mg/kg AMX (Figure 3G). Statistical analysis also showed that the nanoencapsulation and incorporation of DHA resulted in a decrease in the effective dose of the antibiotic. The CA-AMX-DHA group showed significantly higher antimicrobial activity at 10 mg/kg than CA-AMX formulations or free AMX in the same concentrations (*p =* 0.007 and 0.0002, respectively). The antibacterial activity of CA-DHA-AMX was almost the same at the concentrations tested. These results are interesting, and it could be hypothesized that the incorporation of DHA in CA NPs could reduce the effective dose of AMX.

### Bacterial binding and mucoadhesive properties of composite NPs

**Figure 4:**
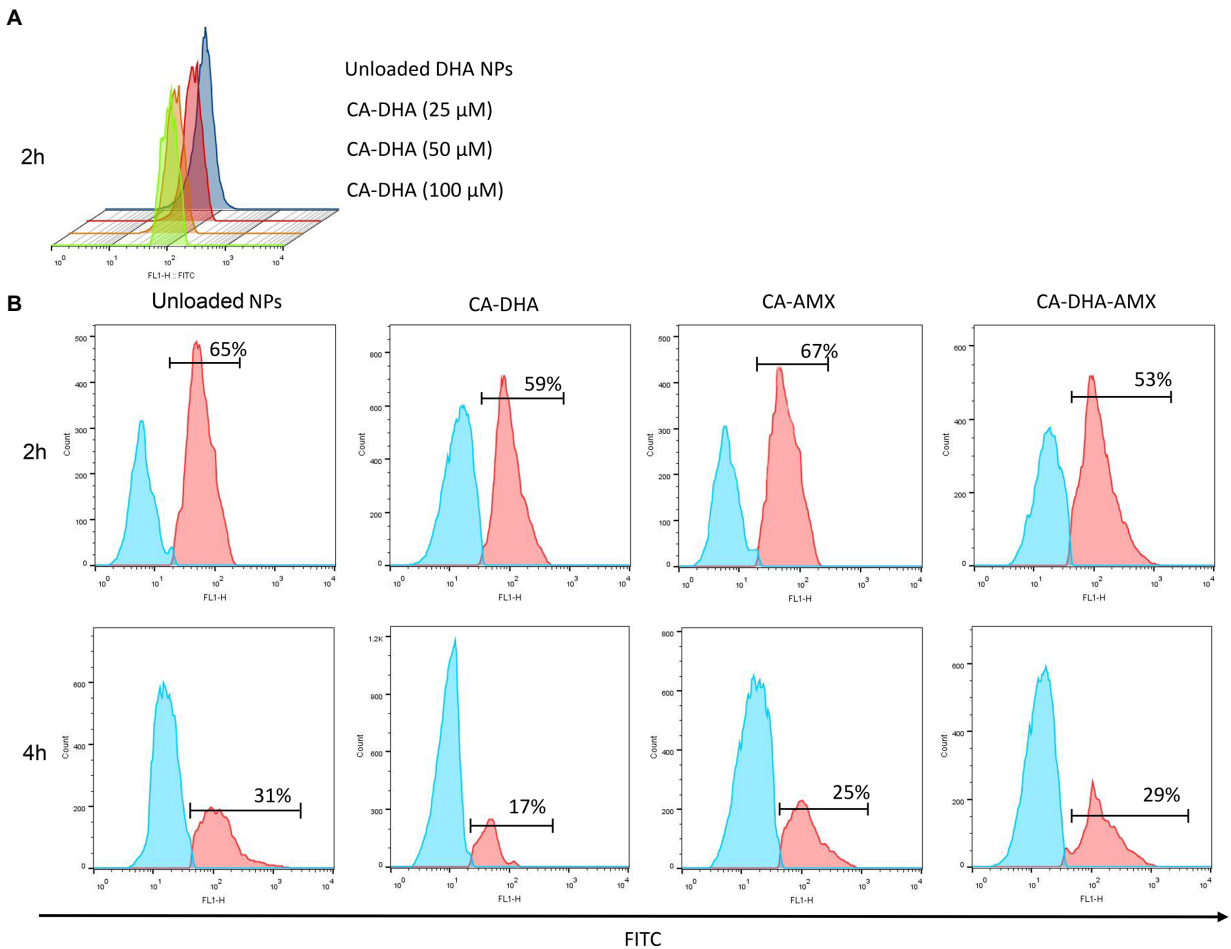
The bacterial binding capacity of composite NPs. **(A)** Bacterial adhesion of CA-DHA NPs and unloaded NPs at different concentrations of DHA. **(B)** The effect of different formulations of NPs on bacterial adhesion at two different time intervals; 2 h and 4 h.

The CA-composite Bacterial binding was studied at different concentrations of DHA. Figure 4A shows that the unloaded NPs exhibited higher adhesion and DHA concentration is negatively correlated with bacterial adhesion. Due to the presence of anionic substances such as lipopolysaccharide groups on the surface of bacteria, this result was expected, as these substances tend to interact with the cationic parts of the chitosan molecules (Gafri et al., 2019). This results in strong interactions with the cationic NH_2_ groups of the protonated chitosan under acidic conditions, which are reduced by DHA (Figures 1E,G). To further investigate this issue, we compared the values of each formulation. In contrast to DHA, AMX showed no effect on bacterial adhesion (Figure 4B). A previous study demonstrated that chitosan-based NPs possessed high mucoadhesive properties and the chitosan amino groups interacted with sialic acids in the mucosa in a pH-dependent manner (Mukhopadhyay et al., 2015).

**Figure 5:**
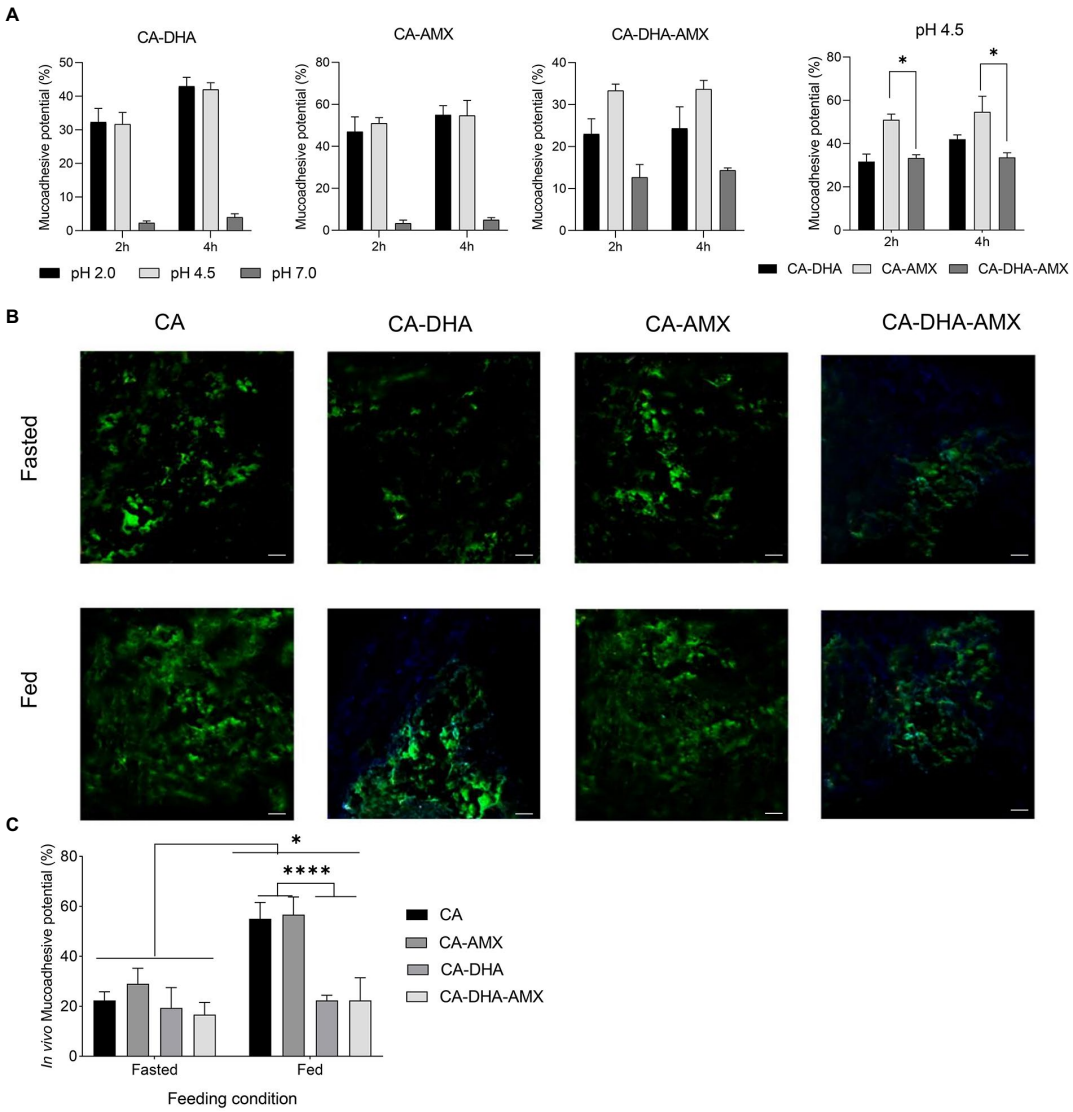
The mucoadhesion potential of composite NPs. **(A)** The *in vitro* assessment of mucoadhesive potential. Results of the unloaded NPs were similar to CA-AMX (data not shown). **(B)** *In vivo* mucoadhesive activity was evaluated by fluorescence microscopy under two distinct diet conditions; fasted (2 h before feeding) and fed (2 h after feeding, scale bar 500 μM). **(C)** The *in vivo* mucoadhesive activity was performed under two feeding conditions, fasting and feeding. Data are presented as mean ± SEM (*n* = 3).

Our results showed that DHA reduced mucoadhesive activity in a dose-dependent manner at all concentrations. We found that the mucoadhesive activity increased when DHA was removed from the formulation, especially at pH 4.5 (*p =* 0.014) (Figure 5A). The mucoadhesive activity was highest in acidic condition (2.5 and 4.5) than pH = 7.5 for different formulation (Figure 5A). The positive charge of chitosan under acidic conditions interacted strongly electrostatically with the negative charge of sialic acid on mucin. As a cationic polysaccharide, chitosan contains primary amino and hydroxyl groups in its repeating units. Primary amino groups carry a positive charge when protonated, which may facilitate electrostatic interactions with negatively charged epithelial cells. The electrostatic interactions are lost when these amino groups are deprotonated at a pH of >6.5. On the other hand, the net charge of mucin becomes more negative with increasing pH. At a pH of 2.5, H+ is abundant in the gastric niche, masking the negative charge of mucin, which creates a competitive effect against chitosan (Sogias et al., 2008; Ways et al., 2018). At pH = 7.5, mucin appears to have a greater net negative charge than at pH = 2.5, while chitosan appears to be more deprotonated and has fewer positive charges than at acidic pH. However, it was observed that the composite NPs interacted electrostatically with mucin molecules at optimal pH values (pH > 4.5).

The mucoadhesive potential was significantly higher in fed than in fasting condition. The acidity of the stomach in the fasting condition (2 h before feeding) and the fed condition (2 h after feeding) is different: 2.5 and 3.0–4.5, respectively. The results showed that the mucoadhesive potential was significantly higher in the fed condition than in the fasting condition (Figure 5C). The mucoadhesive potential of the composite NPs was different at pH = 2.5 and pH = 4.5, although this difference was not significant, but the mucoadhesive potential was higher at pH 4.5 than the others. It was also found that the composite NPs containing DHA exhibited lower mucoadhesive capacity, especially under feeding conditions (*p =* 0.029) (Figures 5B,C). In contrast, a previous study showed that curcumin-loaded NPs with lipid components such as polyvinyl alcohol and polyethylene glycol were more mucoadhesive (Chanburee and Tiyaboonchai, 2017). Chitosan likely quenched DHA in our study.

### *Helicobacter pylori* eradication and ulcer healing

**Figure 6:**
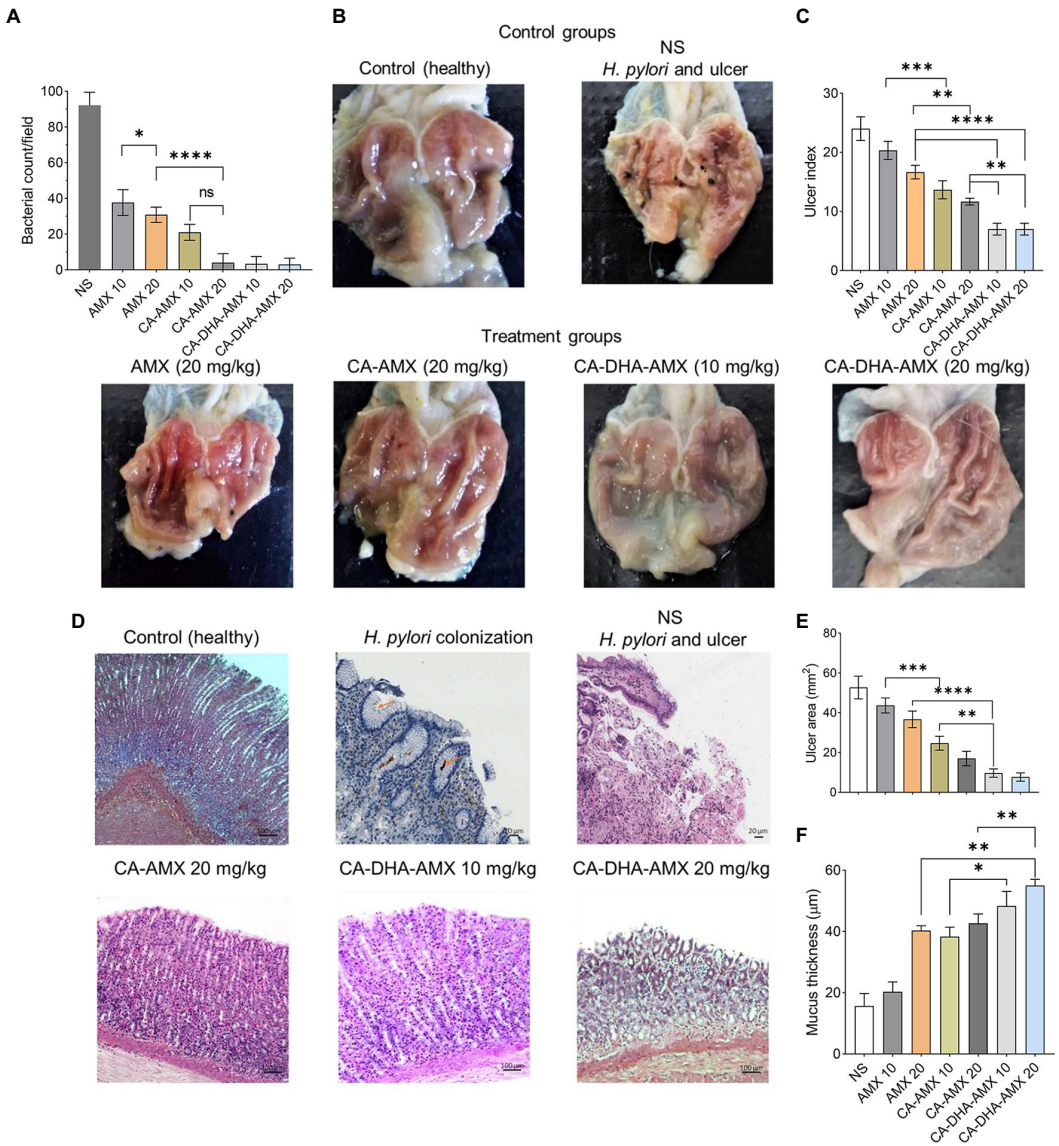
A histopathological evaluation of ASA-induced ulcers in rats infected with *H. pylori*. **(A)** The bacterial count of biopsies in treatment groups at day 14. **(B)** The phenotypic assessment of the rat stomach in different examined groups at day 14. **(C–F)** The analysis of ulcer index, histopathological features, ulcer area, and mucosa thickness of treated groups compared with control (untreated group). Data are presented as mean ± SEM (*n* = 3).

In histopathological studies, NPs incorporated with AMX and DHA were more effective in eradicating *H. pylori*. On day 21, *H. pylori* were found to be completely eradicated with CA-DHA-AMX (10 and 20 mg/kg). We found that DHA significantly increased the efficacy of AMX in the encapsulated formulations. AMX (10 mg/kg) in the encapsulated form significantly increased eradication rates compared with AMX powder 20 mg/kg (*p =* 0.0184) (Figure 6A). On the other hand, an equally high eradication rate was observed with CA-AMX (20 mg/kg) and CA-DHA-AMX (10 and 20 mg/kg). It is suggested that the membrane proteins changed and the permeability and accessibility of the antibiotic led to a significant decrease in the effective dose (Henostroza et al., 2022).

Figure 6B shows the phenotypic healing of ASA-induced ulcers. The ulcers were completely healed by day 14. The inflammation decreased during the administration of CA-DHA compared with AMX (20 mg/kg). CA-DHA-AMX at 10 mg/kg exhibited the same healing effect as 20 mg/kg and showed a higher healing effect than AMX powder (20 mg/kg) (Figure 6B). As shown in Figure 6C, CA-DHA-AMX demonstrated significantly higher ulcer healing activity than CA-AMX (*p =* 0.009). In this study, DHA was found to accelerate the healing of ulcers induced by ASA.

Histological examination of the gastric epithelium confirmed *H. pylori* infection (Figure 6D). Microscopically, we measured the length and thickness of the ulcer (40×, 5 sections/sample). Our results showed that in the CA-DHA-AMX groups with 10 mg/kg and 20 mg/kg AMX, the ulcer areas were significantly decreased compared with the other groups (Figure 6E). The presence of DHA in combination with 10 mg/kg AMX significantly reduced ulcer area compared with AMX powder (20 mg/kg) and the CA-AMX group at 10 mg/kg (*p =* 0.003 and < 0.0001, respectively) (Figure 6E). The encapsulation of AMX also significantly improved the ulcer healing effect, and the mucus thickness was similar between AMX powder (20 mg/kg) and CA-AMX (10 mg/kg) (Figure 6F). Accordingly, the incorporation of AMX with DHA-loaded NPs seemed to be beneficial. This result is consistent with the findings of Anandan et al. study suggesting that chitin and chitosan may be antiulcerogenic (Anandan et al., 2004). The pathological evaluation showed that CA-DHA-AMX reduced inflammatory and hemorrhagic conditions (Figure 6D). CA-DHA-AMX (20 mg/kg) significantly increased mucosal thickness compared with CA-AMX and AMX powder (*p =* 0.0054 and = 0.0011, respectively) (Figures 6D,F). The summary of physicochemical and biological activity of the different formulations is shown in ST. 2.

**Figure 7:**
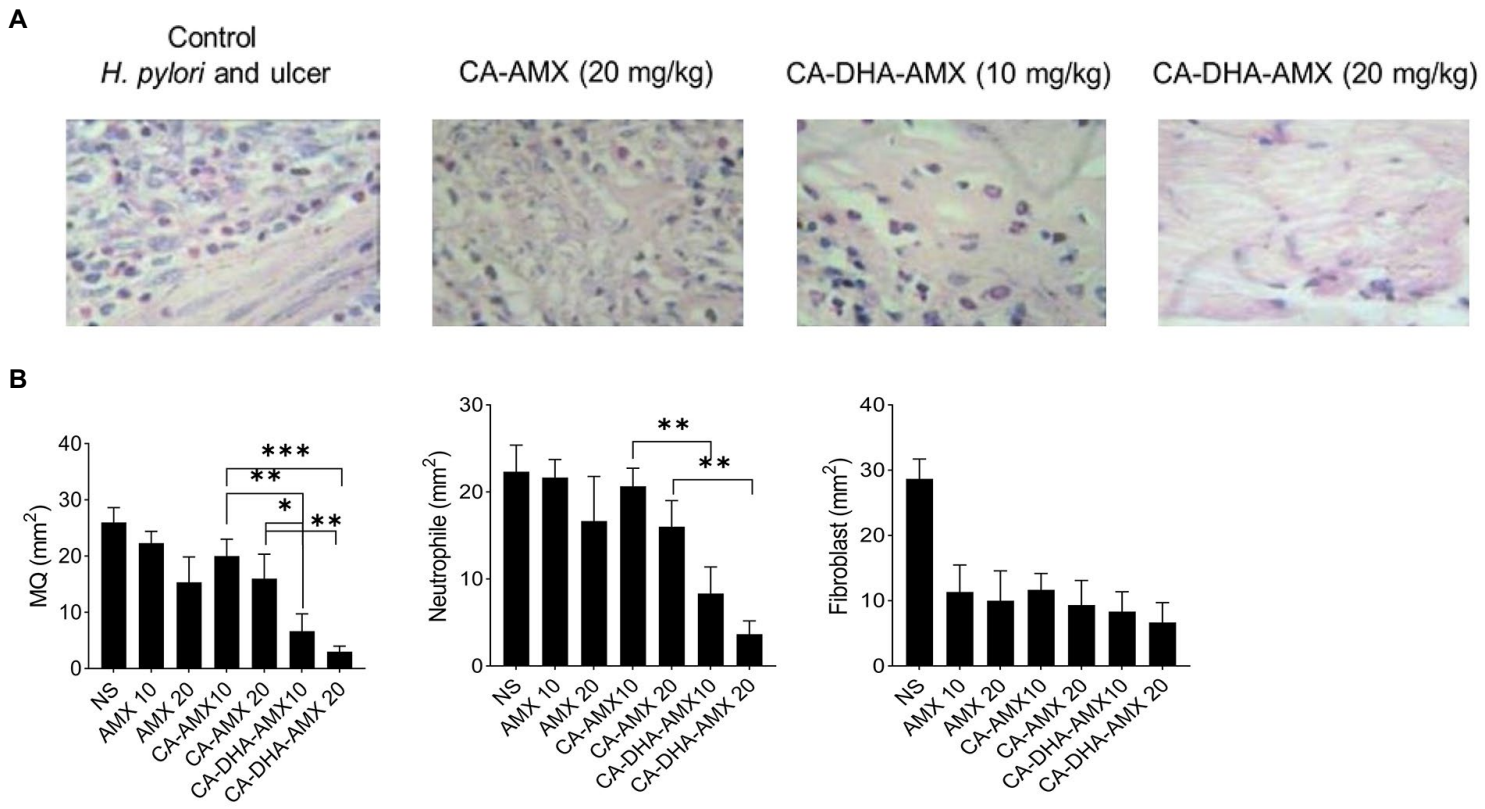
The distribution of macrophage (MQ), neutrophil and fibroblast cells. **(A)** The microscopic distribution of macrophage, neutrophil and fibroblast cells in four different group untreated group, CA-AMX (20 mg/kg), CA-DHA-AMX (10 mg/kg), and CA-DHA, AMX (20 mg/kg) at 14. Hematoxylin and Eosin staining. **(B)** The comparison of macrophage, neutrophil and fibroblast cells at day 14 among all treated groups. Data are presented as mean ± SEM (*n* = 3).

The macroscopic and microscopic evaluation of each group showed that inflammation decreased in the presence of DHA in different formulations (Figures 6B,D). The infiltration of immune cells was reduced after treatment with a formulation containing DHA (Figures 6D, 7A,B). Comparison of macrophages (MQ), neutrophils, and fibroblast cells at days 3 and 14 revealed that DHA in the formulation was associated with a significant decrease in innate immune response cells. The presence of DHA (CA-DHA-AMX groups) significantly decreased the number of MQ and neutrophils at day 14 compared with the absence of DHA (CA-AMX groups) (Figures 7A,B). As recently reported by Pineda-Pea et al., DHA has been shown to have anti-inflammatory and antioxidant properties that can alleviate indomethacin-induced gastric ulcers. Compared to the control group, they found that DHA significantly reduced neutrophil infiltration (Pineda-Peña et al., 2018).

**Figure 8:**
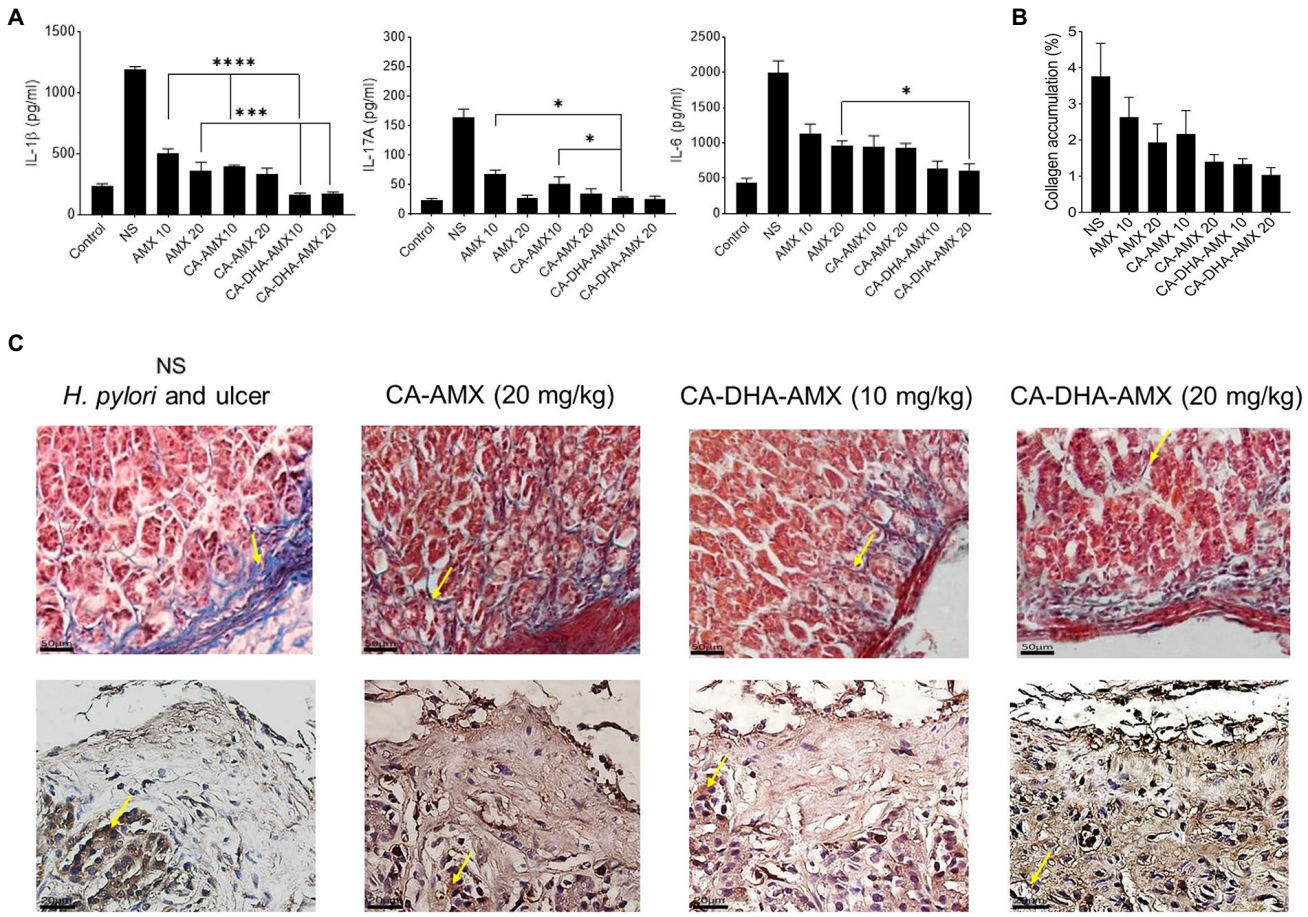
The histopathological evaluation and the pro-inflammatory cytokine production of gastric biopsies. **(A)** The levels of pro-inflammatory cytokines including IL-1β, IL-6, and IL-17A was conducted using ELISA method. **(B)** The collagen accumulation among different treated groups. **(C)** Masson’s trichrome staining was used to evaluate the accumulation of collagen (yellow arrow), the blue and red are responsible for collagen and muscle fibers. Immunohistochemical staining for Collagen I (yellow arrow), high accumulation of collagen I in glands and endothelia observed in untreated group, while the accumulation was reduced in treated groups, the lowest accumulation was observed in CA-DHA-AMX (20 mg/kg). Data are presented as mean ± SEM (*n* = 3).

Pro-inflammatory cytokines such as IL -6, IL -1β, and IL -17A were significantly lower in the formulations containing DHA than in those without. ELISA results showed that the presence of DHA decreased IL -1β in different formulations. In the CA-DHA-AMX (10 mg/kg) group, the concentration of IL -1β was significantly higher than in the formulations that did not contain it. The CA-DHA-AMX groups were compared with the AMX powder and CA-AMX groups, and the results indicated that DHA was responsible for the anti-inflammatory effect of the composite NPs (Figure 8A). The histopathological examination of the examined groups revealed that the collagen accumulation was different (Figure 8B). As demonstrated by immunohistochemical staining for collagen I, CA-AMX (20 mg/kg) accumulated more collagen I than CA-DHA-AMX (20 mg/kg) (Figures 8B,C). In a study by Motawee et al., it was found that chronic administration of DHA significantly reduced the expression of H+/K + -ATPase gene and the enzyme activity of COX −2 while improving the gastric ulcer index, percent ulcer protection, and significantly reducing the expression of gastric GSH, CCK, and e- NOS genes and significantly reducing the expression of gastric GSH, CCK, and e- NOS genes (Motawee et al., 2022). As reported in another study by Serini et al., resveratrol-based solid lipid nanoparticles containing DHA showed anti-inflammatory properties on keratinocytes with a decrease in the expression of IL -1β, IL -6, and MCP-1 (Serini et al., 2019). The results of our study show that chitosan nanoparticles are a reliable means of delivering drugs to the niches of the stomach. Although the addition of DHA reduces the mucoadhesive properties of CA-DHA-AMX NPs, it increases drug entrapment and shows high antibacterial activity and anti-inflammatory effects.

## Conclusion

The development of drugs containing acid-sensitive antibiotics such as AMX is difficult with conventional gastric retention formulation techniques. The development of a therapeutically effective gastroprotective formulation of AMX, which has both excellent buoyancy and a suitable release pattern, could allow the targeting of drugs to specific sites in the stomach. Our developed system had none of the disadvantages of a single-dose formulation but offered the advantage of the ease of preparation and sustained release of the drug over an extended period. A simple method of encapsulating AMX in DHA-loaded CA NPs can be used for biocidal effects against *H. pylori*, with a reduced effective dose of the antibiotic. As a mucoadhesive carrier, chitosan-based nanoparticles are an effective way to deliver acid-sensitive antibiotics such as AMX. The encapsulation of AMX significantly increased its antibacterial activity compared to single AMX, and incorporation of DHA decreased the effective dose. The DHA also decreased the effective dose of AMX in the encapsulated form by increasing its entrapment, which may be due to the modification of bacterial cell walls and its antibacterial activity. The incorporation of DHA into CA-AMX composite NPs enhanced the antibacterial activity *in vivo* and accelerated the healing of gastric ulcers, which could be attributed to the DHA -mediated dissolution of bacterial cell membrane, macrophage-dependent clearance, and anti-inflammatory effects of DHA. The *in vivo* anti-*H. pylori* effects of DHA may also be due to its immunomodulatory activities that elicit biocidal effects on *H. pylori*.

## Data availability statement

The original contributions presented in the study are included in the article/Supplementary material, further inquiries can be directed to the corresponding author.

## Ethics statement

The animal study was reviewed and approved by all animal experiments were performed according to a protocol approved by the Ethics Committee of Ilam Medical University (approval number: R.MEDILAM. REC.1400.133). Written informed consent was obtained from the owners for the participation of their animals in this study.

## Author contributions

MH: conceptualization, visualization, and supervision. MH and MA: methodology and validation. SK and VK: investigation and data curation. SK, NS and MA: writing – original draft preparation. ME-S and BN: writing – reviewing and editing. All authors contributed to the article and approved the submitted version.

## Conflict of interest

The authors declare that the research was conducted in the absence of any commercial or financial relationships that could be construed as a potential conflict of interest.

## Publisher’s note

All claims expressed in this article are solely those of the authors and do not necessarily represent those of their affiliated organizations, or those of the publisher, the editors and the reviewers. Any product that may be evaluated in this article, or claim that may be made by its manufacturer, is not guaranteed or endorsed by the publisher.

## References

[ref1] Anandan R. Nair P. G. Mathew S. (2004). Anti-ulcerogenic effect of chitin and chitosan on mucosal antioxidant defence system in HCl-ethanol-induced ulcer in rats. J. Pharm. Pharmacol. 56, 265–269. doi: 10.1211/0022357023079, PMID: 15005886

[ref2] Arora S. Gupta S. Narang R. K. Budhiraja R. D. (2011). Amoxicillin loaded chitosan–alginate polyelectrolyte complex nanoparticles as mucopenetrating delivery system for *H. pylori*. Sci. Pharm. 79, 673–694. doi: 10.3797/scipharm.1011-05, PMID: 21886911 PMC3163361

[ref3] Bhattacharya A. Ghosal S. Bhattacharya S. K. (2006). Effect of fish oil on offensive and defensive factors in gastric ulceration in rats. Prostaglandins Leukot. Essent. Fatty Acids 74, 109–116. doi: 10.1016/j.plefa.2005.11.001, PMID: 16352428

[ref4] Casillas-Vargas G. Ocasio-Malave C. Medina S. Morales-Guzman C. Del Valle R. G. Carballeira N. M.. (2021). Antibacterial fatty acids: an update of possible mechanisms of action and implications in the development of the next-generation of antibacterial agents. Prog. Lipid Res. 82:101093. doi: 10.1016/j.plipres.2021.101093, PMID: 33577909 PMC8137538

[ref5] Chanburee S. Tiyaboonchai W. (2017). Mucoadhesive nanostructured lipid carriers (NLCs) as potential carriers for improving oral delivery of curcumin. Drug Dev. Ind. Pharm. 43, 432–440. doi: 10.1080/03639045.2016.1257020, PMID: 27808665

[ref6] Chandrasekaran M. Kim K. D. Chun S. C. (2020). Antibacterial activity of chitosan nanoparticles: a review. PRO 8:1173. doi: 10.3390/pr8091173

[ref7] Chang S. H. Hsieh P. L. Tsai G. J. (2020). Chitosan inhibits helicobacter pylori growth and urease production and prevents its infection of human gastric carcinoma cells. Mar. Drugs 18:542. doi: 10.3390/md18110542, PMID: 33138146 PMC7692773

[ref8] Chang P. K. Tsai M. F. Huang C. Y. Lee C. L. Lin C. Shieh C. J.. (2021). Chitosan-based anti-oxidation delivery Nano-platform: applications in the encapsulation of DHA-enriched fish oil. Mar. Drugs 19:470. doi: 10.3390/md19080470, PMID: 34436309 PMC8400499

[ref9] Chen E. P. Mahar Doan K. M. Portelli S. Coatney R. Vaden V. Shi W. (2008). Gastric pH and gastric residence time in fasted and fed conscious cynomolgus monkeys using the bravo® pH system. Pharm. Res. 25, 123–134. doi: 10.1007/s11095-007-9358-5, PMID: 17612796

[ref10] Choi I. J. Kim C. G. Lee J. Y. Kim Y. I. Kook M. C. Park B.. (2020). Family history of gastric cancer and helicobacter pylori treatment. N. Engl. J. Med. 382, 427–436. doi: 10.1056/NEJMoa1909666, PMID: 31995688

[ref11] Coraça-Huber D. C. Steixner S. Wurm A. Nogler M. (2021). Antibacterial and anti-biofilm activity of omega-3 polyunsaturated fatty acids against periprosthetic joint infections-isolated multi-drug resistant strains. Biomedicine 9:334. doi: 10.3390/biomedicines9040334, PMID: 33810261 PMC8065983

[ref12] Correia M. Michel V. Matos A. A. Carvalho P. Oliveira M. J. Ferreira R. M.. (2012). Docosahexaenoic acid inhibits helicobacter pylori growth in vitro and mice gastric mucosa colonization. PLoS One 7:e35072. doi: 10.1371/journal.pone.0035072, PMID: 22529974 PMC3328494

[ref13] Correia M. Michel V. Osório H. El Ghachi M. Bonis M. Boneca I. G.. (2013). Crosstalk between helicobacter pylori and gastric epithelial cells is impaired by docosahexaenoic acid. PLoS One 8:e60657. doi: 10.1371/journal.pone.0060657, PMID: 23577140 PMC3618039

[ref14] Cui Z. Xiang Y. Si J. Yang M. Zhang Q. Zhang T. (2008). Ionic interactions between sulfuric acid and chitosan membranes. Carbohydr. Polym. 73, 111–116. doi: 10.1016/j.carbpol.2007.11.009

[ref15] Dixon S. J. Lemberg K. M. Lamprecht M. R. Skouta R. Zaitsev E. M. Gleason C. E.. (2012). Ferroptosis: an iron-dependent form of nonapoptotic cell death. Cells 149, 1060–1072. doi: 10.1016/j.cell.2012.03.042, PMID: 22632970 PMC3367386

[ref16] Friedman A. J. Phan J. Schairer D. O. Champer J. Qin M. Pirouz A.. (2013). Antimicrobial and anti-inflammatory activity of chitosan–alginate nanoparticles: a targeted therapy for cutaneous pathogens. J. Investig. Dermatol. 133, 1231–1239. doi: 10.1038/jid.2012.399, PMID: 23190896 PMC3631294

[ref17] Gafri H. F. S. Zuki F. M. Aroua M. K. Hashim N. A. (2019). Mechanism of bacterial adhesion on ultrafiltration membrane modified by natural antimicrobial polymers (chitosan) and combination with activated carbon (PAC). Rev. Chem. Eng. 35, 421–443. doi: 10.1515/revce-2017-0006

[ref18] Graham D. Y. Lu H. Shiotani A. (2021). Vonoprazan-containing helicobacter pylori triple therapies contribution to global antimicrobial resistance. J. Gastroenterol. Hepatol. 36, 1159–1163. doi: 10.1111/jgh.15252, PMID: 32918832

[ref19] Henostroza M. A. B. Tavares G. D. Yukuyama M. N. De Souza A. Barbosa E. J. Avino V. C.. (2022). Antibiotic-loaded lipid-based nanocarrier: a promising strategy to overcome bacterial infection. Int. J. Pharm. 621:121782. doi: 10.1016/j.ijpharm.2022.121782 35489605

[ref20] Henriques P. C. Costa L. M. Seabra C. L. Antunes B. Silva-Carvalho R. Junqueira-Neto S.. (2020). Orally administrated chitosan microspheres bind helicobacter pylori and decrease gastric infection in mice. Acta Biomater. 114, 206–220. doi: 10.1016/j.actbio.2020.06.035, PMID: 32622054

[ref21] Hu Y. Du Y. Yang J. Kennedy J. F. Wang X. Wang L. (2007). Synthesis, characterization and antibacterial activity of guanidinylated chitosan. Carbohydr. Polym. 67, 66–72. doi: 10.1016/j.carbpol.2006.04.015

[ref22] Ji H. G. Piao J. Y. Kim S. J. Kim D. H. Lee H. N. Na H. K.. (2016). Docosahexaenoic acid inhibits helicobacter pylori-induced STAT3 phosphorylation through activation of PPARγ. Mol. Nutr. Food Res. 60, 1448–1457. doi: 10.1002/mnfr.201600009, PMID: 27079734

[ref23] Kim J. Wang T. C. (2021). Helicobacter pylori and gastric cancer. Gastrointest. Endosc. Clin. 31, 451–465. doi: 10.1016/j.giec.2021.03.003 34053633

[ref24] Krzyżek P. Paluch E. Gościniak G. (2020). Synergistic therapies as a promising option for the treatment of antibiotic-resistant helicobacter pylori. Antibiotics 9:658. doi: 10.3390/antibiotics9100658, PMID: 33007899 PMC7599531

[ref25] Kuo C. J. Lee C. H. Chang M. L. Lin C. Y. Lin W. R. Su M. Y.. (2021). Multidrug resistance: the clinical dilemma of refractory helicobacter pylori infection. J. Microbiol. Immunol. Infect. 54, 1184–1187. doi: 10.1016/j.jmii.2021.03.006, PMID: 33840604

[ref26] Li J. Wu Y. Zhao L. (2016). Antibacterial activity and mechanism of chitosan with ultra high molecular weight. Carbohydr. Polym. 148, 200–205. doi: 10.1016/j.carbpol.2016.04.025, PMID: 27185132

[ref27] Luo D. Guo J. Wang F. Sun J. Li G. Cheng X.. (2009). Preparation and evaluation of anti-helicobacter pylori efficacy of chitosan nanoparticles in vitro and in vivo. J. Biomater. Sci. Polym. Ed. 20, 1587–1596. doi: 10.1163/092050609X12464345137685, PMID: 19619399

[ref28] Motawee M. E. Hassan F. Damanhory A. M. Mohie P. M. Khalifa M. M. Elbastawisy Y. M. (2022). Possible protective effect of each of Omega-3 PUFA and leptin on indomethacin-induced gastric ulcer in rats with type II DM. Bull. Egypt. Soc. Physiol. Sci. 42, 329–343. doi: 10.21608/besps.2022.121674.1119

[ref29] Mukhopadhyay P. Chakraborty S. Bhattacharya S. Mishra R. Kundu P. P. (2015). pH-sensitive chitosan/alginate core-shell nanoparticles for efficient and safe oral insulin delivery. Int. J. Biol. Macromol. 72, 640–648. doi: 10.1016/j.ijbiomac.2014.08.040, PMID: 25239194

[ref30] Pacheco N. Naal-Ek M. G. Ayora-Talavera T. Shirai K. Román-Guerrero A. Fabela-Morón M. F.. (2019). Effect of bio-chemical chitosan and gallic acid into rheology and physicochemical properties of ternary edible films. Int. J. Biol. Macromol. 125, 149–158. doi: 10.1016/j.ijbiomac.2018.12.060, PMID: 30529349

[ref31] Pineda-Peña E. A. Martínez-Pérez Y. Galicia-Moreno M. Navarrete A. Segovia J. Muriel P.. (2018). Participation of the anti-inflammatory and antioxidative activity of docosahexaenoic acid on indomethacin-induced gastric injury model. Eur. J. Pharmacol. 818, 585–592. doi: 10.1016/j.ejphar.2017.11.015, PMID: 29154839

[ref32] Qin Y. Lao Y.-H. Wang H. Zhang J. Yi K. Chen Z.. (2021). Combatting helicobacter pylori with oral nanomedicine. J. Mater. Chem. B 9, 9826–9838. doi: 10.1039/D1TB02038B, PMID: 34854456

[ref33] Rahaiee S. Hashemi M. Shojaosadati S. A. Moini S. Razavi S. H. (2017). Nanoparticles based on crocin loaded chitosan-alginate biopolymers: antioxidant activities, bioavailability and anticancer properties. Int. J. Biol. Macromol. 99, 401–408. doi: 10.1016/j.ijbiomac.2017.02.095, PMID: 28254570

[ref34] Seabra C. L. Nunes C. Gomez-Lazaro M. Correia M. Machado J. C. Goncalves I. C.. (2017). Docosahexaenoic acid loaded lipid nanoparticles with bactericidal activity against helicobacter pylori. Int. J. Pharm. 519, 128–137. doi: 10.1016/j.ijpharm.2017.01.014, PMID: 28088639

[ref35] Serini S. Cassano R. Facchinetti E. Amendola G. Trombino S. Calviello G. (2019). Anti-irritant and anti-inflammatory effects of DHA encapsulated in resveratrol-based solid lipid nanoparticles in human keratinocytes. Nutrients 11:1400. doi: 10.3390/nu11061400, PMID: 31234344 PMC6627705

[ref36] Shah S. C. Iyer P. G. Moss S. F. (2021). AGA clinical practice update on the Management of Refractory Helicobacter pylori infection: expert review. Gastroenterology 160, 1831–1841. doi: 10.1053/j.gastro.2020.11.059, PMID: 33524402 PMC8281326

[ref37] Sogias I. A. Williams A. C. Khutoryanskiy V. V. (2008). Why is chitosan mucoadhesive? Biomacromolecules 9, 1837–1842. doi: 10.1021/bm800276d, PMID: 18540644

[ref38] Spósito L. Fortunato G. C. de Camargo B. A. F. Ramos M. A. D. S. Souza M. P. C. D. Meneguin A. B.. (2021). Exploiting drug delivery systems for oral route in the peptic ulcer disease treatment. J. Drug Target. 29, 1029–1047. doi: 10.1080/1061186X.2021.1904249, PMID: 33729081

[ref39] Suzuki H. Matsuzaki J. (2018). Gastric cancer: evidence boosts helicobacter pylori eradication. Nat. Rev. Gastroenterol. Hepatol. 15, 458–460. doi: 10.1038/s41575-018-0023-8, PMID: 29713024

[ref40] Takahashi T. Imai M. Suzuki I. Sawai J. (2008). Growth inhibitory effect on bacteria of chitosan membranes regulated with deacetylation degree. Biochem. Eng. J. 40, 485–491. doi: 10.1016/j.bej.2008.02.009

[ref41] Ways T. M. M. Lau W. M. Khutoryanskiy V. V. (2018). Chitosan and its derivatives for application in mucoadhesive drug delivery systems. Polymers 10:267. doi: 10.3390/polym10030267, PMID: 30966302 PMC6414903

