# Supplementary material for: Amoxicillin-docosahexaenoic acid encapsulated chitosan-alginate nanoparticles as a delivery system with enhanced biocidal activities against Helicobacter pylori and improved ulcer healing
Source: Front Microbiol. 2023 Feb 9;14:1083330. doi: 10.3389/fmicb.2023.1083330 (PMC9948253; doi:10.3389/fmicb.2023.1083330)
Supplement: Supplementary file 1 [file Data_Sheet_1.docx]

**Supplementary file**

**Amoxicillin-Docosahexaenoic acid encapsulated Chitosan-Alginate nanoparticles as a delivery system with enhanced biocidal activities against *Helicobacter pylori* and improved ulcer healing**

Saeed Khoshnood^1^, Babak Negahdari^2^, Mohamed El-Shazly^3^, Mohd Azmuddin Abdullah^4^, Mohammad Hossein Haddadi^1*^

^1^ Clinical Microbiology Research Centre, Ilam University of Medical Sciences, Ilam, Iran

^2^ Department of Medical Biotechnology, School of Advanced Technologies in Medicine, Tehran University of Medical Sciences, Tehran, Iran

^3^ Pharmacognosy Department, Faculty of Pharmacy, Ain-Shams University, Cairo, Egypt.

^4^ Department of Toxicology, Advanced Medical and Dental Institute, Universiti Sains Malaysia, Bertam Campus, Kepala Batas 13050, Penang, Malaysia

***Corresponding author:** Dr. Mohammad Hossein Haddadi, Assistant Professor in Medical Biotechnology,

Email: haddadi-m@medilam.ac.ir, [haddadi841@gmail.com](mailto:haddadi841@gmail.com). Tel: 08432227101, +989188417822. P.O. Box 6939177143.

**Section 1. Materials and Methods**

The material chitosan (95% deacetylation, high molecular weight; 310000-325000 Da) purchased from Hexir Co. Iran. Sodium alginate (Mw = 20 kDa, G content of 65–70%), Fluorescein isothiocyanate in 10 mg vial (FITC; Invitrogen F-1906). Sheep blood obtained from parsteb (Iran). Fetal bovine serum (FBS) purchased from DENAzist Asia (Iran). Docosahexaenoic acid (DHA), trimethoprim, vancomycin, amoxicillin (AMX), polymyxin B, and Cellulose membrane purchased from Sigma Aldrich (Gillingham, UK). Gaspack C obtained from Merck (USA). Collagen I was obtained from (PA-26264, Thermofisher, USA). Enzyme-linked immunosorbent assay (ELISA) kits were purchased from Bioassay Technology Laboratory (China).

**Section 2. Composite NPs preparation**

We prepared chitosan-based NPs containing both DHA and AMX by employing the ionic gelation method together with alginate. A polycationic solution was prepared by dissolving (20 gr) of chitosan powder in acetic acid (0.1, 0.5, and 1.0% v/v) under magnetic stirring and continuous sonication for 60 min. During the homogenization process, the solution was passed through a filter with a pore size of 1 μm. Preparation of the polyanionic solution was accomplished by dispersing the alginate powder (20 gr) in distilled water and stirring mechanically. The pH of the solution was adjusted to 4.2 with 2 N NaOH. The final solutions were stirred at 50 °C until the solution was completely homogenized (without any aggregation).

We prepared three different concentrations of DHA (1.0, 1.5, and 2.0% v/v). Homogenization was performed in a water bath at 50 °C for 90 min. DHA, AMX and chitosan solutions were gradually dropped into the alginate solution under ice bath conditions for 20 minutes during homogenization and then placed in the freezer at -20 °C for 2 min.

Finally, ultrasonic treatment was performed using an ultrasonic device in the ice bath for 4 min with a sequence of 0.7 s sonication and 0.3 s rest, resulting in a homogeneous suspension. The resulting particles were collected by centrifugation at 10,000 × g at -20 °C for 30 min. Unloaded DHA and AMX were removed by washing the plate several times with DDW. Finally, We prepared CA-DHA NPs in different formulations, chitosan (0.1, 0.5, and 1.0% v/v), DHA (0.0, 0.5, 1.5, and 2.0 v/v), and AMX (40, 60, and 100 mg/ml), *in vivo* study was conducted to test CA-DHA, CA-AMX, and CA-DHA-AMX NPs by following concentration of components: CA (1.0% v/v), DHA (2.0% v/v), and AMX in two concentration; 10 and 20 mg/kg.

**Section.3 Physicochemical characteristics**

***Scanning electron microscopy analysis***

Surface morphology was determined by SEM analysis. The CA -based NPs were mounted on an aluminum stub and coated with gold (30 mM, 8 Pa) for 10 s using a sputter coater (Polaron). The surface of the composite scaffolds was analyzed using SEM (Tokyo, Japan). The gold-coated samples were observed with an accelerating voltage of 10 Kv.

***Fourier transform infrared sp******ectroscopy (FTIR)***

FTIR was used to identify functional groups and evaluate the localization of AMX and DHA in CA NPs. All components were analyzed by FTIR, including chitosan, alginate, DHA, AMX and CA-DHA-AMX NPs. The NPs were first freeze-dried to study the interaction between the amino groups of the components. The NPs were loaded onto potassium bromide (KBr) disks and then analyzed using an FTIR spectrometer (Shimadzu Scientific Instruments, USA). The samples were scanned from 400 to 4000 cm-1.

***X-ray diffraction***

X-ray diffraction (XRD) was used to determine the crystallinity and structure of the composite NPs. X-ray diffraction analysis was performed using a Philips X'Pert MPD diffractometer (Philips, The Netherlands) equipped with Cu Kα radiation (λ = 1,540 Å) at 30 mA and 40 kV. The samples were placed on a sample holder and XRD scans were measured in the 2 θ range from 10° to 80° at a rate of 2°/min.

***Atomic force microscopy***

The surface morphology of NPs and particle size were determined by atomic force microscopy (AFM).

***Zeta Potential***

The zeta potential of the composite NPs was evaluated by Zeta potential analyzer (Horiba Jobin Jyovin) in buffer solution with different pH values. For each composite nanoparticle, three replicate samples were measured at different pH values and ±SD for triplicate samples was reported as the mean diameter.

***Swelling index***

Swelling was studied on composite NPs in different pH media. The composite NPs were dried and weighed (1 g) before immersion in distilled water as medium for 2 h. The composite NPs were then dried and weighed. The stability of the composite NPs in different media (pH 2.5, 4.0, and 5.5) was determined using a spectrophotometer at a wavelength of 500 nm. The swelling index was determined in the composite NPs using the following formula:

$\% SI=\frac{\left( Ws-Wd \right)}{wd} \times100$

Where SI indicates the swelling index and "Ws" and "Wd" represent the weight of the swollen and dry composite NPs, respectively.

***Drug content***

Stock solutions of AMX were prepared by dissolving AMX in methanol at a concentration of 10 μg/ml. Finally, working solutions were prepared in concentration ranges of 5, 10, 15, 20, and 25 μg/ml with methanol.

Twenty milligrams of NPs were accurately weighed, compressed, and crushed by sonication. The released contents were washed with PBS. Tween 80 was used to increase the solubility of AMX. The resulting solution was made up to a volume of 100 ml in a 100 ml volumetric flask containing PBS. The solution was filtered through a 0.45 µm membrane filter to collect the NP debris. The filtered solution was analyzed for AMX at 272 nm and for DHA at 205 nm using a UV/Vis spectrophotometer (JENWAY/6105). Measurements were performed in triplicate for 60 mg/ml AMX and 100 µM DHA. The drug content was expressed as drug entrapment (DE %) and calculated using the following equation:

$$\%DE =\frac{M1}{M0} \times100$$

"M1" and "M0" represent the amount of AMX or DHA in the NPs and the amount of drug used in the formulation, respectively.

**Section. 4 Bacterial isolation and antibacterial activity**

The clinically isolated *H. pylori* was obtained from a gastric biopsy specimen from a patient with gastric ulceration (H.12.5). H.12.5 showed amoxicillin and metronidazole sensitivity and clarithromycin resistance phenotype (data not published). Isolation was performed on Brucella agar supplemented with sheep blood, fetal bovine serum (FBS) 5%, trimethoprim (5 μg/mL), vancomycin (10 μg/mL), and polymyxin B (0.25 μg/mL) under microaerophilic conditions (Gaspack C, Merck, USA). A stock solution of the bacteria was prepared in Columbia broth medium and adjusted to 10^9^ CFU/ml. Under microaerophilic conditions at 37˚C, 10 µl of the bacteria suspended in culture medium (without antibiotics) was added to the 96-well plate and stirred continuously. The antibacterial activity of CA, DHA, CA-DHA, CA-AMX and CA - DHA-AMX was determined by calculating the growth inhibition rate. At intervals of 6, 12, and 24 h, the bacterial growth ratio was measured using a spectrophotometer at a wavelength of 600 nm. AMX and medium served as negative and positive control groups, respectively. Unloaded CA NPs were examined at different concentrations (0.1, 0.5, and 1%). DHA was tested at three different concentrations (25, 50 and 100 µM) against *H. pylori* at the time intervals of 6, 12 and 24 h.

Based on the number of CFU/g stomach, the antibacterial activity was determined *in vivo* at day 7. The biopsy sample is gently homogenized and suspended in 2 ml PBS and centrifuged. The supernatant was removed and the plate was suspended in 0.5 ml PBS and cultured on enriched blood agar. The plate was incubated at 37°C for 24 h. To *in vivo* antibacterial activity, we administrated pure chitosan (50 g/kg), free- DHA (1 ml, 100 µM), AMX, CA-AMX, CA-DHA, and CA-DHA-AMX.

**Section. 5 Nanoparticle-*****H. pylori* interaction**

***Preparation of FITC-labelled NPs***

The isothiocyanate group of FITC and the amino group of chitosan react to form FITC-labeled NPs (FITC-NPs). Briefly, 30 mg of NPs was dissolved in 15 mL of 1 M HCl and a pH of 6.9 was adjusted with 1 M NaOH. The solution above was added with FITC dissolved in alcohol (2.1 mg). After 24 hours of stirring at room temperature, the mixture was dialyzed with distilled water using a cellulose membrane for 2 days, and lyophilized. The entire procedure was conducted in the dark. A UV/VIS spectrophotometer was used to determine the FITC-labelled NPs content by comparison of the visible absorption at the maximum wavelength of about 491 nm between the conjugate NPs and FITC.

The suspensions of FITC-composite NPs (50 g/ml) were incubated with PBS containing 1*10^7^ CFU/ml of *H. pylori* for 30 minutes at 37°C. Flow cytometry was used to determine the adherence of NPs to bacterial cells. The adherence rate of NPs to bacterial cell surfaces was measured using flow cytometry at 2 and 4 h intervals.

1. Arora, S., et al., *Amoxicillin loaded chitosan–alginate polyelectrolyte complex NPs as mucopenetrating delivery system for H. pylori.* Scientia pharmaceutica, 2011. 79(3): p. 673-694.

Supplementary Figure 1


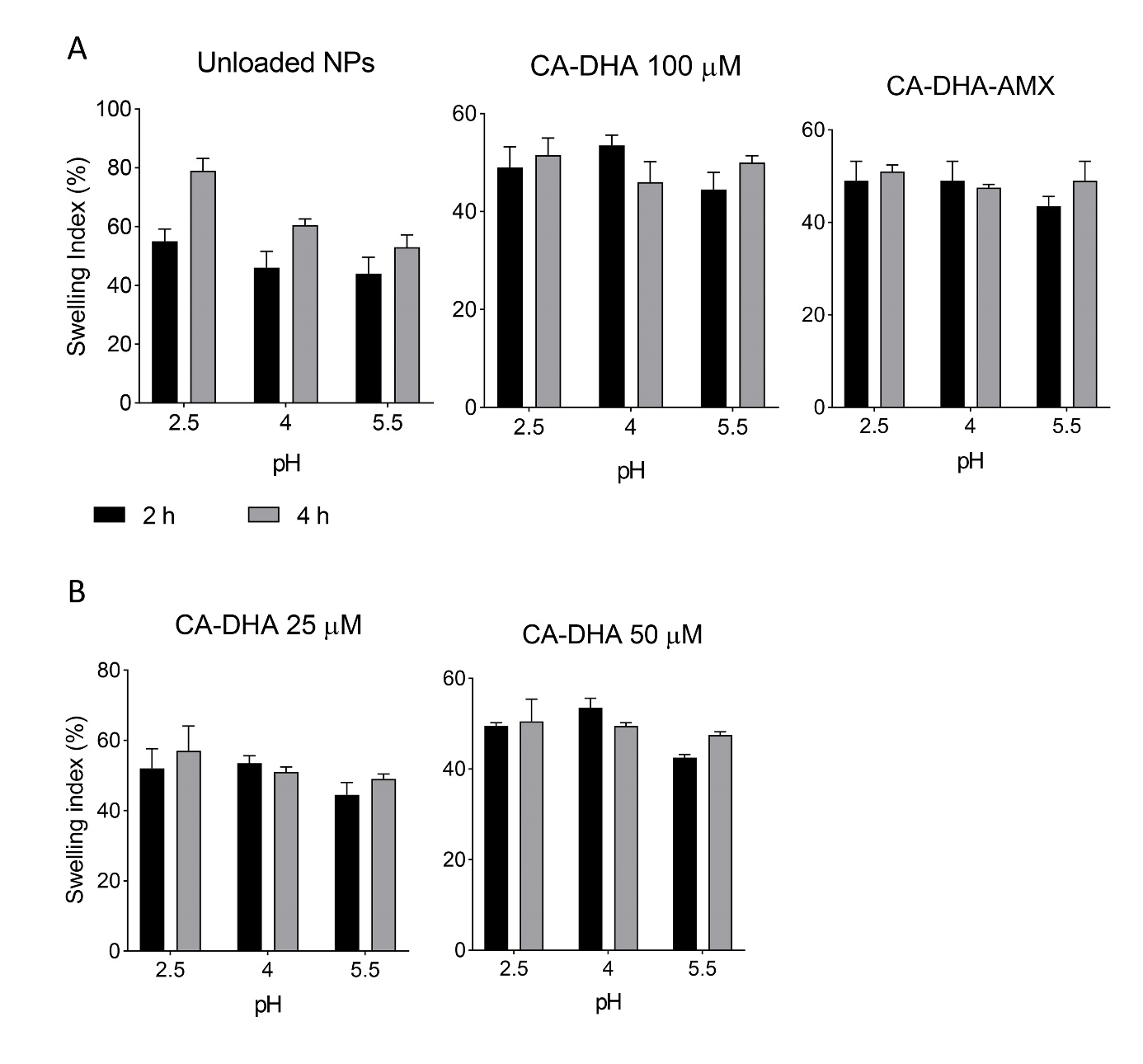
**Figure 1.** (A) The swelling index of composite NPs. (B) The swelling index of composite NPs containing different concentrations of DHA.

Supplementary Table 1. The experimental groups of infected rats and treatment options.

| Groups | Name | Treatment  Drugs (mg/kg) |
| --- | --- | --- |
| *In vivo* antibacterial activity* | | |
| AG1 | Chitosan | 50 g/kg |
| AG2 | DHA | 1 ml (100 μm) |
| AG3 | CA-DHA | (1% v/v)- (2%) |
| AG4 | NS | Normal saline (1ml) |
| AG5 | AMX-10 | AMX= 10 mg/kg |
| AG6 | AMX-20 | AMX= 20 mg/kg |
| AG7 | CA-AMX-10 | AMX= 10 mg/kg |
| AG8 | CA-AMX-20 | AMX= 20 mg/kg |
| AG9 | CA-DHA-AMX-10 | AMX= 10 mg/kg |
| AG10 | CA-DHA-AMX-20 | AMX= 20 mg/kg |
| *In vivo H. pylori* eradication and gastric ulcer healing** | | |
| G1 | Control | Healthy rat |
| G2 | NS | Normal saline (1ml) |
| G3 | AMX-10 | AMX= 10 mg/kg |
| G4 | AMX-20 | AMX= 20 mg/kg |
| G5 | CA-AMX-10 | AMX= 10 mg/kg |
| G6 | CA-AMX-20 | AMX= 20 mg/kg |
| G7 | CA-DHA-AMX-10 | AMX= 10 mg/kg |
| G8 | CA-DHA-AMX-20 | AMX= 20 mg/kg |

NS: Normal Saline; AMX: Amoxicillin; CA: Chitosan/Alginate; DHA: Docosahexaenoic acid.

**In vivo* antibacterial activity was evaluated at day 7 post-infection.

** *In vivo* *H. pylori* eradication and gastric ulcer healing were assessed at day 14 post-infection, while the recurrence of H. pylori was evaluated at day 21 post-infection.

| Treatment groups | Particle size (nm) | Zeta potential (pH = 6.8 ) | Swelling index** | In vitro Mucoadhesive potential* | Bacterial adhesion** | Growth inhibition (10^3^) | Ulcer area (mm^2^) | Ulcer thickness (μm) |
| --- | --- | --- | --- | --- | --- | --- | --- | --- |
| AMX 10 | - | - | - | - | - | 383±49 | 43±3.1 | 20±3.2 |
| AMX 20 | - | - | - | - | - | 293±62 | 36±4.1 | 40±1.5 |
| CA-AMX 10 | 600±92 | +21±3 mv | 60±3 | 57±8 | 67% | 368±71 | 24±3.5 | 38±3.05 |
| CA-AMX 20 | 600±92 | +23± 3 mv | 60±3 | 57±8 | 67% | 188±56 | 17±3.6 | 42±3.6 |
| CA-DHA-AMX 10 | 350±110 | +8 ± 2 | 57±5 | 22±7 | 53% | 207±55 | 9.6±2.08 | 48±4.7 |
| CA-DHA-AMX 20 | 350±110 | +8 ± 2 | 57±5 | 22±7 | 53% | 168±60 | 7.6±2.8 | 55±2 |

Supplementary Table 2. The summary of physicochemical and biological activity of various formulation

*pH= 4.5, after 4h incubation.

** pH= 5.5.
